# Supplementary material for: Exploring Community-Based Suicide Prevention in the Context of Rural Australia: A Qualitative Study
Source: Int J Environ Res Public Health. 2023 Feb 1;20(3):2644. doi: 10.3390/ijerph20032644 (PMC9915251; doi:10.3390/ijerph20032644)
Supplement: Supplementary file 1 [file ijerph-20-02644-s001.zip › ijerph-2096179-Supplementary File S2.pdf]

'Preventing Suicide in Australia's Rural Youth: Towards Best Practice Guidelines for Community-Based Programs'

Key Informant Expert Interviews – Topic Guide

Experience

1. Please tell me about your experience with suicide prevention, and specifically rural youth suicide prevention?
2. How do you define community-based suicide prevention? What is your experience in this area?

Rural youth suicide and its prevention at a community-level

3. What strengths do rural communities provide in terms of delivering community-based suicide prevention initiatives, to youth, and in general? (i.e. closely connected, local knowledge)
4. What benefits are there for the community in delivering these initiatives?
5. What existing services or organisations are best-placed to lead community-based suicide prevention initiatives? (i.e. Neighbourhood houses, councils, other community groups)
6. Are there any challenges currently impacting on communities to deliver effective rural youth suicide prevention initiatives? (i.e. COVID, service access/provision, access to means)

Delphi study

7. In terms of community-based suicide prevention, what areas of youth suicide prevention are most prominent in rural areas?
8. What supports or information are needed to assist community-based organisations to undertake suicide prevention initiatives?
9. What other distinct areas of rural youth suicide prevention should be included as statements in the Delphi study? (i.e. access to means, data and evidence, training types, organisational support)

Guidelines

10. What are the key components that you feel should be included in '*Best Practice Guidelines for Youth Suicide Prevention in Rural Australian Communities'* (the Guidelines)?
11. What key areas stand out in terms of rural youth suicide prevention that you feel should be highlighted in the Guidelines?

Additional topics

12. Are there any key documents (for example policy or research studies) that should be considered within the development of the Delphi study or Guidelines?
13. Who do you feel should be involved in Delphi study and development of the Guidelines?
14. And lastly, do you have anything else you would like to add?
